# Ammonia mediates cortical hemichannel dysfunction in rodent models of chronic liver disease

**DOI:** 10.1002/hep.29031

**Published:** 2017-03-07

**Authors:** Anna Hadjihambi, Francesco De Chiara, Patrick S. Hosford, Abeba Habtetion, Anastassios Karagiannis, Nathan Davies, Alexander V. Gourine, Rajiv Jalan

**Affiliations:** ^1^ UCL Institute for Liver and Digestive Health, Division of Medicine, UCL Medical School, Royal Free HospitalRowland Hill StreetLondonUnited Kingdom; ^2^ Centre for Cardiovascular and Metabolic Neuroscience, Neuroscience, Physiology and PharmacologyUniversity College LondonLondonUnited Kingdom; ^3^ Neurocentre Magendie, INSERM U1215, Bordeaux, FranceUniversity of BordeauxBordeauxFrance

## Abstract

The pathogenesis of hepatic encephalopathy (HE) in cirrhosis is multifactorial and ammonia is thought to play a key role. Astroglial dysfunction is known to be present in HE. Astrocytes are extensively connected by gap junctions formed of connexins, which also exist as functional hemichannels allowing exchange of molecules between the cytoplasm and the extracellular milieu. The astrocyte‐neuron lactate shuttle hypothesis suggests that neuronal activity is fueled (at least in part) by lactate provided by neighboring astrocytes. We hypothesized that in HE, astroglial dysfunction could impair metabolic communication between astrocytes and neurons. In this study, we determined whether hyperammonemia leads to hemichannel dysfunction and impairs lactate transport in the cerebral cortex using rat models of HE (bile duct ligation [BDL] and induced hyperammonemia) and also evaluated the effect of ammonia‐lowering treatment (ornithine phenylacetate [OP]). Plasma ammonia concentration in BDL rats was significantly reduced by OP treatment. Biosensor recordings demonstrated that HE is associated with a significant reduction in both tonic and hypoxia‐induced lactate release in the cerebral cortex, which was normalized by OP treatment. Cortical dye loading experiments revealed hemichannel dysfunction in HE with improvement following OP treatment, while the expression of key connexins was unaffected. *Conclusion*: The results of the present study demonstrate that HE is associated with central nervous system hemichannel dysfunction, with ammonia playing a key role. The data provide evidence of a potential neuronal energy deficit due to impaired hemichannel‐mediated lactate transport between astrocytes and neurons as a possible mechanism underlying pathogenesis of HE. (Hepatology 2017;65:1306‐1318)

**Abbreviations**
4‐CIN: α‐cyano‐4‐hydroxycinnamic acid
aCSF: artificial cerebrospinal fluid
ALF: acute liver failure
ANOVA: analysis of variance
BDL: bile duct ligation
CBF: carboxyfluorescein
CBX: carbenoxolone
HA: hyperammonemic
HE: hepatic encephalopathy
MCT: monocarboxylate transporter
NPPB: 5‐nitro‐2‐(3‐phenylpropylamino) benzoic acid
OP: ornithine phenylacetate

Hepatic encephalopathy (HE) is a serious neuropsychiatric complication that is associated with liver dysfunction and is diagnosed when other known brain disorders are excluded.^1^ HE comprises a range of symptoms, including sleep disturbances and alterations in cognitive, behavioral, fine motor, and psychomotor functions, with coma and death occurring at the late stages.^2^ Several hypotheses regarding the pathogenesis of HE have been proposed, and numerous factors have been suggested as key players in HE, including inflammation,^3^ oxidative stress,^4^ impaired brain energy metabolism,^5^ and—most commonly—the neurotoxic effects of ammonia.^6^

Recent evidence has demonstrated that astroglial lactate production and release in cortical cultures and in the somatosensory cortex of anesthetized rats is facilitated in the presence of ammonia.^7^ This appears to be due to acidification of the mitochondrial matrix resulting in a direct inhibition of mitochondrial pyruvate uptake. Increased brain lactate levels have also been reported in hyperammonemic conditions such as in acute liver failure (ALF), which is thought to be due to inhibition of the tricarboxylic acid cycle enzyme α‐ketoglutarate dehydrogenase, suggesting a reduction in oxidative metabolism.^8^ It remains unknown whether significant changes in brain lactate metabolism develop in conditions of long‐term central nervous system exposure to increased ammonia concentrations, such as that seen during chronic liver disease or HE.

Astrocytes, the most numerous glial cells in the central nervous system, are thought to play an important role in HE pathogenesis. The astrocytic dysfunction developing during the progression of the disease could precipitate neuronal pathology, leading to neurological impairment. Astrocytes are extensively connected by gap junctions formed of connexins, which also exist as functional hemichannels allowing effective transfer of ions, metabolic substrates, and signaling molecules across the plasma membrane.^9^ Under normal physiological conditions, hemichannels are either closed^10^ or in a flickering state.^11^ In certain pathological conditions, such as epilepsy and ischemia, significant changes in astroglial structure and function may occur, which are associated with changes in connexin hemichannel function, affecting coupling within the astroglial networks and their communication with other brain cells.^12^ We hypothesized, that in HE, connexin hemichannel dysfunction may contribute to the development of its neurological features.

There is recent evidence that hemichannels may function as a conduit of lactate transport across the membrane.^13^ In this study, we first investigated whether connexin hemichannel expression and hemichannel‐mediated release of lactate are altered in animal models of HE. Ornithine phenylacetate (OP, OCR‐002; Ocera Therapeutics, CA) has been shown to reduce ammonia levels in animal models of cirrhosis and ALF. OP treatment was found to be associated with a significant reduction in the severity of brain swelling,^14^ improvement in neurophysiological function,^15^ and reduction in intracranial pressure.^16^ Therefore, in this study we applied OP as an experimental ammonia‐lowering treatment. The data obtained demonstrate that, in HE, ammonia mediates cortical hemichannel dysfunction associated with a significant reduction in hemichannel‐mediated lactate release.

## Materials and Methods

All experiments were performed in accordance with the Animals (Scientific Procedures) Act of 1986, which was revised according to the European Directive 2010/63/EU. All animals received humane care according to the criteria outlined in the *Guide for the Care and Use of Laboratory Animals* (National Institutes of Health publication 86‐23; revised 1985).

### ANIMAL MODELS

Male Sprague‐Dawley rats (body weight, 350‐400 g) were obtained from Charles River Laboratories (Kent, UK).

#### Bile Duct Ligation Surgery

Under general anesthesia (5% isoflurane in 100% oxygen for induction, 2% isofluorane in air for maintenance) 30 rats underwent triple ligation of the bile duct (way of a small laparotomy) to induce chronic liver injury and were studied 28 days after surgery.^17^

#### Noncirrhotic Hyperammonemia Condition

Thirty‐two rats were administered a hyperammonemic (HA) diet. The amino acid recipe used for a stock of approximately 100 g was: 15 g leucine, 7.7 g phenylalanine, 7 g glutamate, 10 g alanine, 4.4 g proline, 5.8 g threonine, 11 g aspartate, 5 g serine, 4.8 g glycine, 3.3 g arginine, 9.6 g lysine, 8.4 g histidine, 3 g tyrosine, 1.5 g tryptophan, and 10.6 g valine. 25 g of this mix (mixed 1:5 with standard rodent chow powder) was freshly prepared daily with water in a mash form and rats were given free access to it for 5 days. The recipe approximates the amino acid composition of a rodent haemoglobin,^18^ mimicking the effect of gastrointestinal bleeding, which is known to result in systemic hyperammonemia.^19^

#### OP Treatment

Three weeks after surgery, 24 bile duct ligation (BDL)‐operated rats were given twice‐daily intraperitoneal injections of combined l‐ornithine and phenylacetate (0.3 g/kg; OP) approximately 7 hours apart for 5 days—a regimen that has been shown previously to effectively reduce plasma ammonia concentration.^20^ The rats were studied on day 28 after BDL surgery within 3 hours of the last OP injection.

Blood and brain tissue were collected under terminal isoflurane anesthesia. Plasma biochemistry was performed using a Cobas Integra II system (Roche Diagnostics, West Sussex, UK).

#### Brain Slice Preparation

Rats were sacrificed humanely by way of isoflurane inhalation overdose. After cardiac perfusion with chilled (4 °C) artificial cerebrospinal fluid (aCSF; 124 mM NaCl, 3 mM KCl, 2 mM CaCl_2_, 26 mM NaHCO_3_, 1.25 mM NaH_2_PO_4_, 1 mM MgSO_4_, 10 mM d‐glucose saturated with 95% O_2_, 5% CO_2_, pH 7.5, *P*CO_2_ 35 mmHg), with an additional 9 mM Mg^2+^, the brains were rapidly removed and placed in a bath of chilled (4 °C‐6 °C) aCSF. Coronal cortical slices (300 µm thick) were cut using a vibrating microtome. The slices were recovered in oxygenated (95% O_2_, 5% CO_2_) aCSF at room temperature for 30 minutes.

### WESTERN BLOT ANALYSIS

Proteins (30 μg) extracted from the cortices of five sham‐operated, six BDL, six HA, and five BDL‐OP treated rats were separated by way of sodium dodecyl sulphate‐polyacrylamide gel electrophoresis on a 4%‐12% Bis‐Tris NuPAGE gel (Invitrogen, Scotland, UK) and transferred to nitrocellulose membranes. Membranes were blocked with 5% bovine serum albumin and incubated with antibodies against connexin‐43 (Cell Signaling Technology, Danvers, MA; 1:1000), connexin‐36 (Santa Cruz Biotechnology, Dallas, TX; 1:1000), connexin‐30 (Invitrogen, 1 µg/mL), and connexin‐26 (Thermo Fisher Scientific, Waltham, MA; 1 µg/mL). Detection of actin (Santa Cruz Biotechnology; 1:1000) was used to control for protein loading. Binding of antibody was detected using a horseradish peroxidase–conjugated secondary antibody (goat anti‐rabbit or goat anti‐mouse IgG‐HPR, Santa Cruz Biotechnology; 1:10,000) where appropriate and the SuperSignal Chemiluminescence Substrate for detection of horseradish peroxidase (Pierce, Thermo Fisher Scientific, Waltham, MA). Densitometric analysis was performed using Kodak 1D image analysis software (Kodak, Rochester, NY).

### MEASUREMENTS OF LACTATE RELEASE USING MICROELECTRODE BIOSENSORS

**Figure 1:**
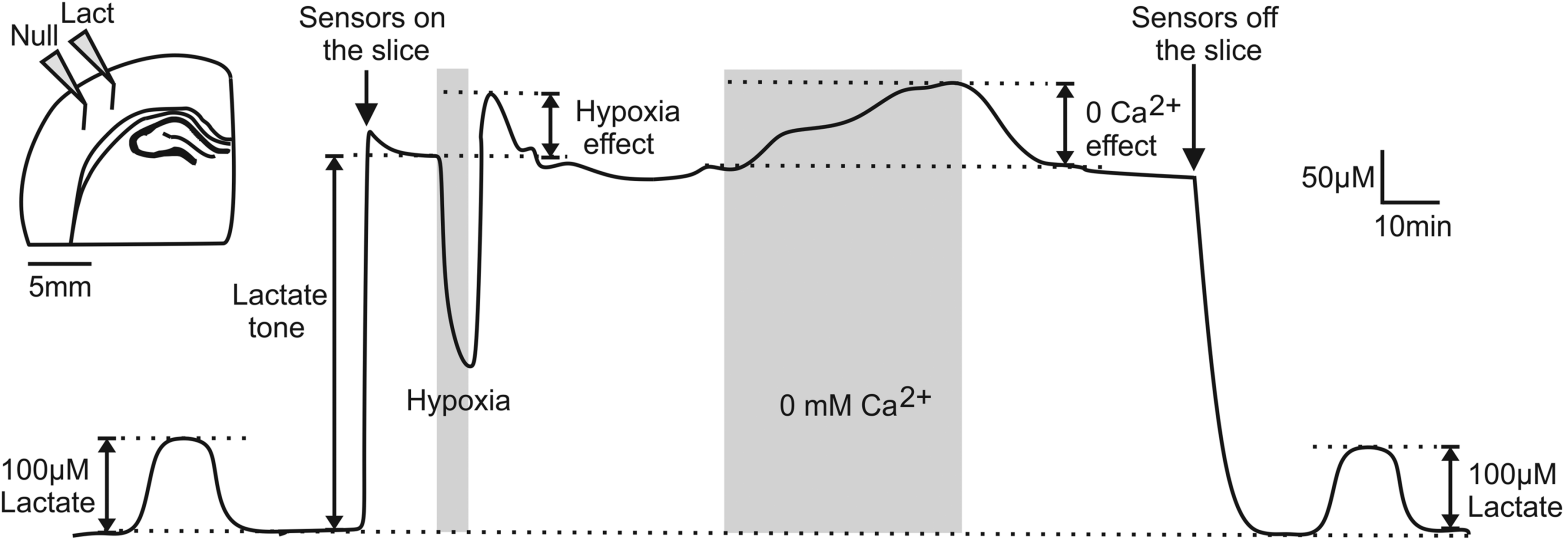
Measuring release of lactate using microelectrode biosensors. Representative example of changes in the net lactate biosensor current (difference in current between lactate and null sensors) during calibration (100 μM lactate), after biosensor placement in direct contact with the surface of the cortical slice (recording tonic lactate release), in response to a hypoxic challenge (perfusion with aCSF saturated with 95% N_2_/5% CO_2_), and in response to lowering extracellular [Ca^2+^]. Peak hypoxia‐induced lactate release is measured upon reoxygenation. Upper left: Schematic drawing of the dual recording configuration of lactate and null (control) biosensors placed on the surface of the brain slice.

Amperometric enzymatic biosensors were obtained from Sarissa Biomedical (Coventry, UK). The design and operation of the biosensors have been described in detail previously.^21^, ^22^ All sensors were operating against a reference electrode (Ag/AgCl) and had a linear response to lactate within the concentration range recorded in this study.^13^, ^22^ Further information on the principle of operation and response time is given in the online data supplement (Supporting Fig. S1A‐C). To control for the release of nonspecific electroactive interferants, a dual recording configuration was used. In every recording, a “null” sensor, lacking enzymes but otherwise identical, was used to measure current changes not associated with lactate oxidase activity, which were then subtracted from the current recorded by the lactate biosensor^23^ (Fig. 1).

The sensors were calibrated directly in the slice chamber immediately before and after every recording by application of 100 μM of lactate (Fig. 1). To convert changes in the biosensor current to changes in lactate concentration, an average of sensor calibrations before and after the recording were used. For each of the recordings, a slice was transferred into the recording chamber and superfused with aCSF at 35 °C (3 mL/min^−1^). Sensors were initially placed in the chamber having no contact with the brain slice. Once a steady‐state baseline was achieved, the sensors were laid flat in direct contact with the cut surface of the cerebral cortex (Fig. 1), revealing tonic lactate release which stabilized within approximately 15 minutes. Hypoxic conditions, known to increase both lactate production due to inhibition of oxidative phosphorylation and the opening probability of connexin hemichannels,^13^, ^24^ were induced for 2‐4 minutes by replacement of oxygen in the medium with nitrogen (perfusion of the chamber with aCSF saturated with 95% N_2_/5% CO_2_).^25^, ^26^ Because detection of lactate by the biosensors requires oxygen (Supporting Fig. S1B),^27^ the effect of hypoxia was determined by measuring the peak lactate release upon reoxygenation (Fig. 1) as described in detail previously.^13^, ^27^ Once the baseline was restored, Ca^2+^‐free aCSF (with the addition of 1 mM ethylene glycol tetraacetic acid) was applied for 20 minutes as the second stimulus known to increase the opening probability of certain membrane channels, including connexin hemichannels.^28^ There is no prior evidence that Ca^2+^‐mediated increases in mitochondrial NADH influence cytosolic NAD^+^/NADH homeostasis and therefore lactate production.^29^ These stimuli were reapplied in the presence of connexin hemichannel blockers carbenoxolone (CBX, 100 µM; Sigma, Poole, UK) or 5‐nitro‐2‐(3‐phenylpropylamino)benzoic acid (NPPB, 200 µM; Sigma). CBX and NPPB have previously been shown to have no effect on lactate biosensor detection system.^13^

### ASSESSMENT OF HEMICHANNEL FUNCTION USING DYE LOADING

For the assessment of hemichannel functionality (effectiveness of channel opening and closing) we used a fluorescent dye carboxyfluorescein (CBF; 376 Da). Connexin hemichannels are permeable to CBF and can act as conduits of CBF transport across the membrane in accord with the concentration gradient of the dye. Cortical slices from sham‐operated, BDL, HA, and BDL‐OP treated rats were exposed to normal aCSF with an addition of CBF (200 μm) for 9 minutes resulting in background connexin‐mediated dye loading, followed by perfusion with Ca^2+^‐free aCSF without CBF for 9 minutes resulting in CBF unloading. Ca^2+^‐free aCSF with CBF was then applied for 4 minutes increasing the permeability of hemichannels and therefore resulting in dye loading. Hypoxic conditions (without CBF) were next applied to unload the slice and also to demonstrate bidirectional permeability of the channels to CBF.^26^ The same hypoxic stimulus was then reapplied in the presence of CBF, resulting in dye loading. After application of each stimulus in the presence of CBF, a further 5‐minute perfusion with aCSF containing CBF was performed, followed by a 10‐minute wash with normal aCSF, enabling the channels to return to their physiological state. Images were taken using MiCAM‐02 imaging system (SciMedia, Costa Mesa, CA). Using ImageJ software, regions of interest were drawn around the areas of the cerebral cortex (∼3 cm^2^) across layers I‐III, and the mean pixel intensities for the regions were calculated. Background fluorescence was subtracted.

### STATISTICAL ANALYSIS

**Figure 2:**
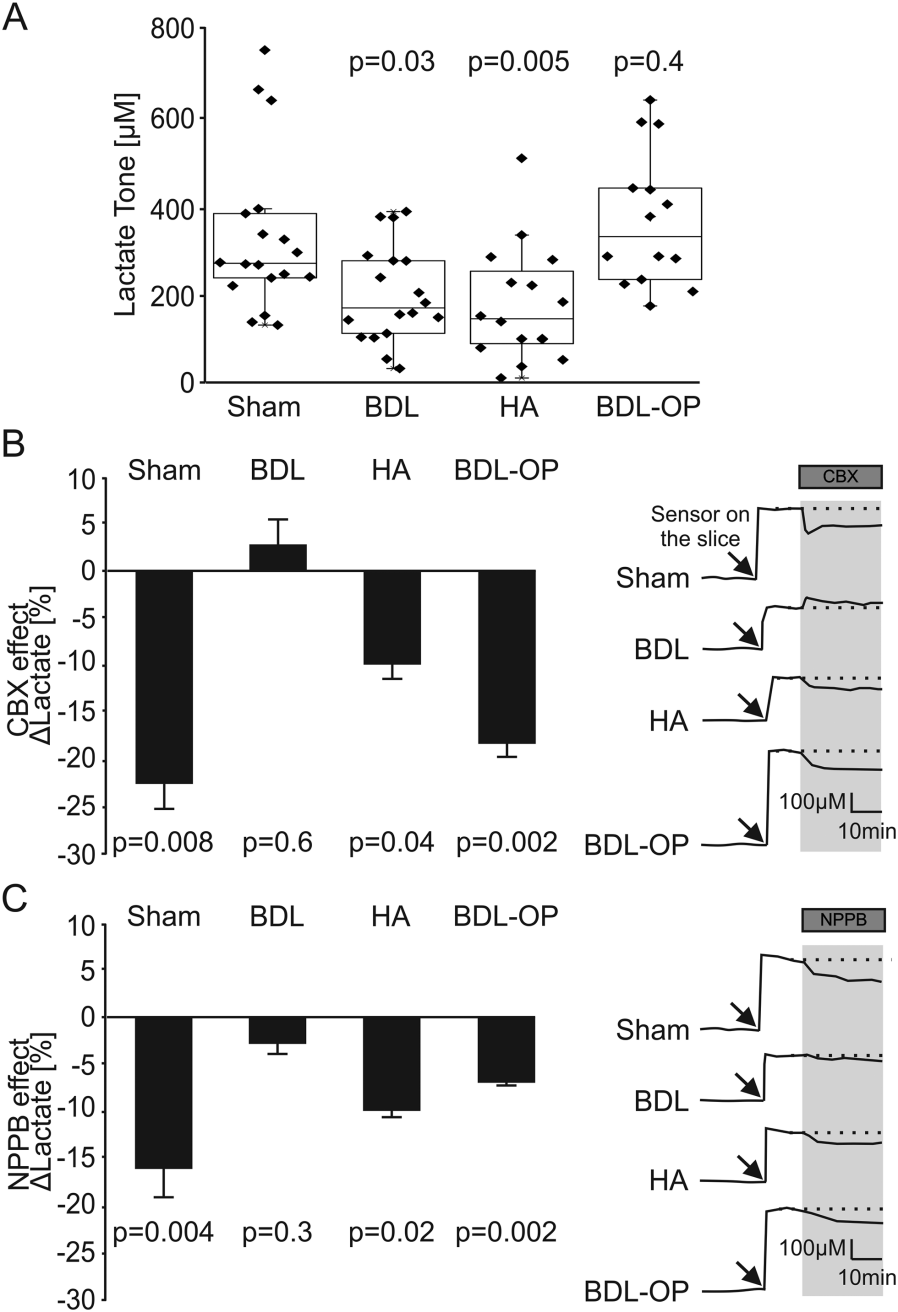
HE is associated with a reduction in hemichannel‐mediated release of lactate in the cerebral cortex. (A) Summary data illustrating tonic release of lactate in cortical slices of sham‐operated, BDL, HA, and BDL‐OP treated rats. *P* values indicate differences from the sham‐operated rats. (B) Left: Summary data illustrating the effect of CBX (100 μM) on tonic release of lactate (expressed as percent change from the baseline) in cortical slices of sham‐operated, BDL, HA, and BDL‐OP treated rats. Right: Representative recordings of lactate biosensor current showing changes in tonic release of lactate in response to CBX application. *P* values indicate differences from the respective baseline. (C) Left: Summary data illustrating the effect of NPPB (200 μM) on tonic release of lactate (expressed as percent change from the baseline) in cortical slices of sham‐operated, BDL, HA, and BDL‐OP treated rats. Right: Representative recordings of lactate biosensor current showing changes in tonic release of lactate in response to NPPB application. *P* values indicate differences from the respective baseline.

**Figure 3:**
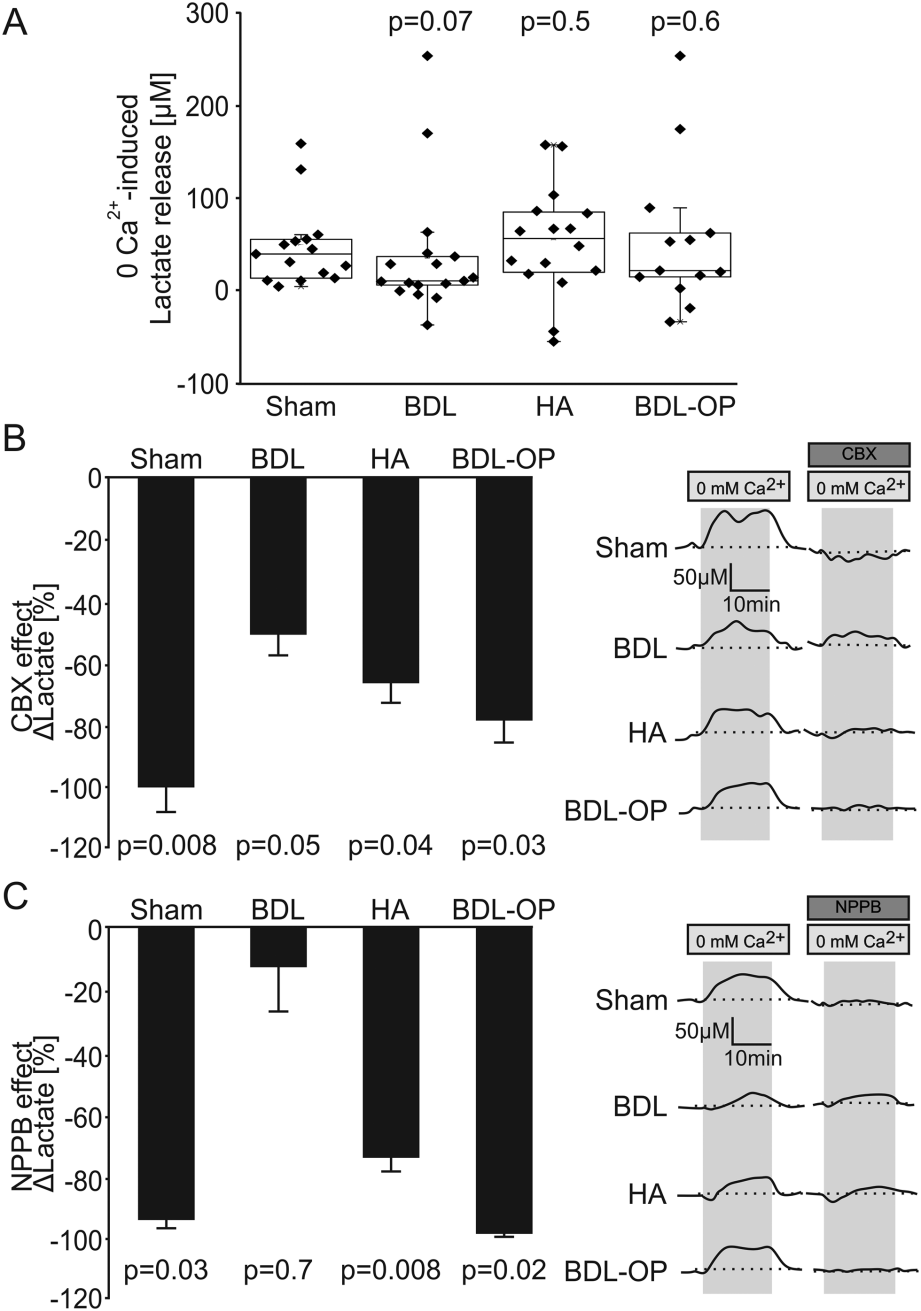
Lactate release in response to lowering extracellular [Ca^2+^]. (A) Summary data illustrating peak changes in lactate release in response to lowering [Ca^2+^]_e_ in cortical slices of sham‐operated, BDL, HA, and BDL‐OP treated rats. *P* values indicate differences from the responses recorded in sham‐operated rats. (B) Left: Summary data illustrating the effect of CBX (100 μM) on the release of lactate facilitated in response to 0 [Ca^2+^]_e_ (expressed as the percentage of the amount of lactate released in response to 0 [Ca^2+^]_e_ in the absence of CBX) in cortical slices of sham‐operated, BDL, HA, and BDL‐OP treated rats. Right: Representative recordings of lactate biosensor current showing the effect of CBX on 0 [Ca^2+^]_e_‐induced release of lactate. *P* values indicate differences between the responses recorded in the absence and presence of CBX. (C) Left: Summary data illustrating the effect of NPPB (200 μM) on the release of lactate facilitated in response to 0 [Ca^2+^]_e_ (expressed as the percentage of the amount of lactate released in response to 0 [Ca^2+^]_e_ in the absence of NPPB) in cortical slices of sham‐operated, BDL, HA, and BDL‐OP treated rats. Right: Representative recordings of lactate biosensor current showing the effect of NPPB on 0 [Ca^2+^]_e_‐induced release of lactate. *P* values indicate differences between the responses recorded in the absence and presence of NPPB.

**Figure 4:**
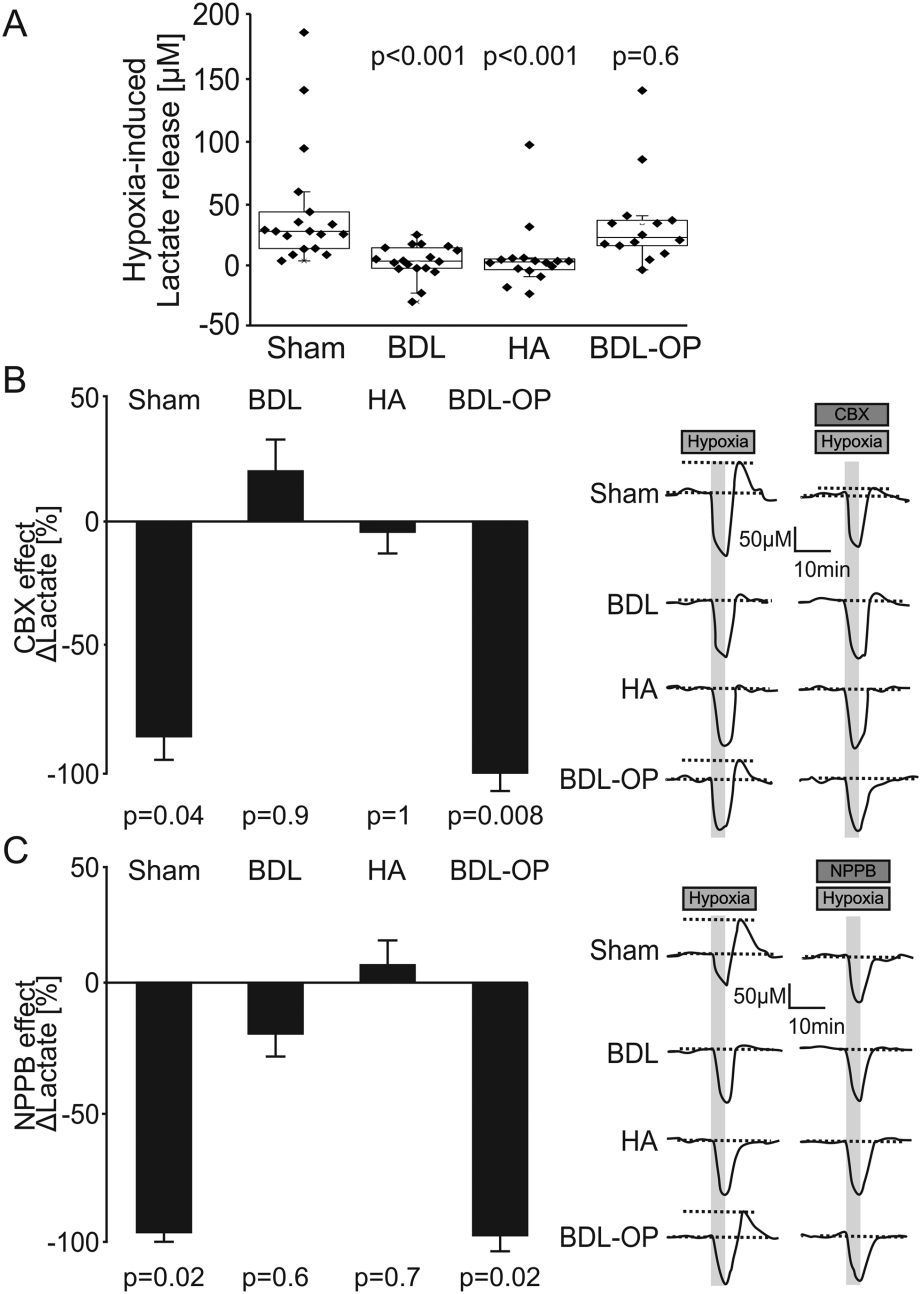
HE is associated with a reduction of hypoxia‐induced release of lactate. (A) Summary data illustrating peak changes in lactate release in response to hypoxia (aCSF saturated with 95% N_2_/5% CO_2_) in cortical slices of sham‐operated, BDL, HA, and BDL‐OP treated rats. *P* values indicate differences from the responses recorded in sham‐operated rats. (B) Left: Summary data illustrating the effect of CBX (100 μM) on the release of lactate facilitated in response to hypoxia (expressed as the percentage of the amount of lactate released in response to hypoxia in the absence of CBX) in cortical slices of sham‐operated, BDL, HA, and BDL‐OP treated rats. Right: Representative recordings of lactate biosensor current showing the effect of CBX on hypoxia‐induced release of lactate. Decrease in O_2_ availability reduces biosensor current followed by a positive signal upon reoxygenation, which is used to estimate hypoxia‐induced lactate release. *P* values indicate differences between the responses recorded in the absence and presence of CBX. (C) Left: Summary data illustrating the effect of NPPB (200 μM) on the release of lactate facilitated in response to hypoxia (expressed as the percentage of the amount of lactate released in response to hypoxia in the absence of NPPB) in cortical slices of sham‐operated, BDL, HA, and BDL‐OP treated rats. Right: Representative recordings of lactate biosensor current showing the effect of NPPB on the hypoxia‐induced release of lactate. *P* values indicate differences between the responses recorded in the absence and presence of NPPB.

Western blot data were normalized using the protocol of LI‐COR Biosciences (Normalization Accuracy for Western Blotting), and group data were compared using two‐way analysis of variance (ANOVA) with a Tukey *post hoc* test. Data obtained using biosensor recordings were analyzed and presented nonparametrically using box and whisker plots (Figs. 2A, 3A, 4A). For comparisons between the experimental groups, a Mann‐Whitney U test was applied. The peak hypoxia‐ or 0 Ca^2+^‐induced lactate releases are presented as changes in release from the baseline (Fig. 1). The effects of connexin blockers are presented as percent changes from the control responses recorded in the absence of the blockers, and a Wilcoxon signed rank test was applied for comparison. The *P* values in Figs. 2B,C, 3B,C, and 4B,C indicate the significance level of differences between the control responses and the responses recorded in the presence of the drugs.

**Figure 5:**
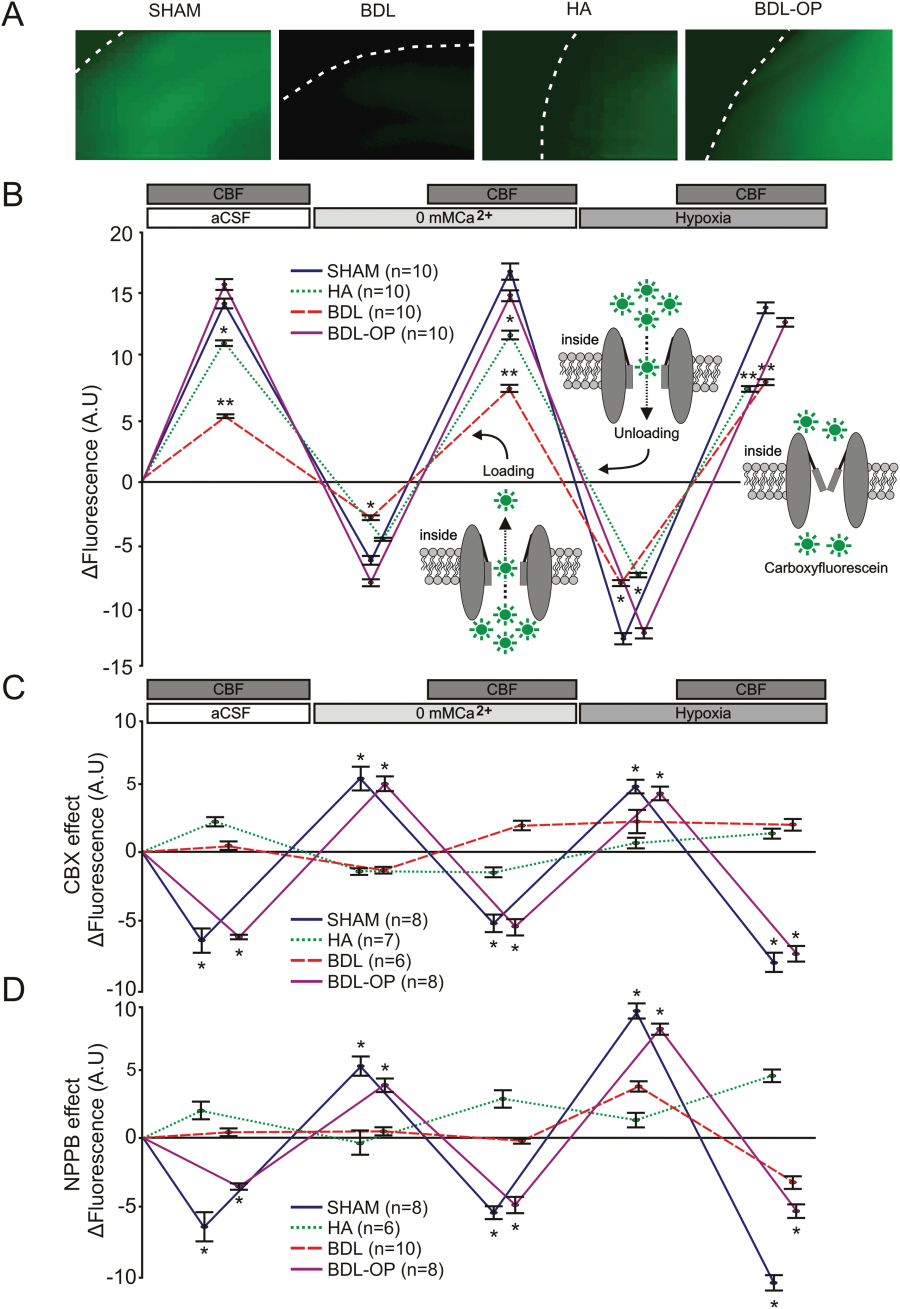
Impaired hemichannel‐mediated dye loading reveals cortical hemichannel dysfunction in HE. (A) Representative images of background loading with CBF dye in cortical slices of sham‐operated, BDL, HA, and BDL‐OP treated rats. White dashed lines depict the edge of each cortical slice. (B) Fluorescence intensity changes in cortical slices of sham‐operated, BDL, HA, and BDL‐OP treated rats in response to 0 [Ca^2+^]_e_ and hypoxia in the absence and presence of CBF in the medium. Application of 0 [Ca^2+^]_e_ aCSF or hypoxia in the presence of CBF resulted in dye loading and an increase in fluorescence, whereas application of these stimuli in the absence of CBF resulted in dye unloading and a decrease in fluorescence. Insets: Schematic drawings of connexin hemichannel mediated dye loading and unloading. *P < 0.05, **P < 0.001 versus sham‐operated animals. (C) Summary data illustrating the effect of CBX (100 μM) on fluorescence intensity changes (ΔFluorescence) in cortical slices of sham‐operated, BDL, HA, and BDL‐OP treated rats induced by 0 [Ca^2+^]_e_ and hypoxia in the absence and presence of CBF in the medium. The data are presented as differences in fluorescence after CBX application compared with the respective fluorescence recorded in the absence of CBX. *P < 0.05 for the effect of CBX on CBF loading and unloading. (D) Summary data illustrating the effect of NPPB (200 μM) on fluorescence intensity changes (ΔFluorescence) in cortical slices of sham‐operated, BDL, HA, and BDL‐OP treated rats induced by 0 [Ca^2+^]_e_ and hypoxia in the absence and presence of CBF in the medium. The data are presented as differences in fluorescence after NPPB application compared with the respective fluorescence recorded in the absence of NPPB. *P < 0.05 for the effect of NPPB on CBF loading and unloading.

Data obtained in dye‐loading experiments were analyzed using two‐way ANOVA (Fig. 5B, data normally distributed) followed by a Tukey *post hoc* test or Wilcoxon signed rank test (Fig. 5C,D, data not normally distributed) as appropriate. The biochemistry data were analyzed using one‐way ANOVA. Data are reported as the mean ± standard error of the mean. Differences with *P* < 0.05 were considered to be statistically significant. Sample sizes were calculated using Gpower 3 v3.1.9.2 (http://www.gpower.hhu.de/en.html)^30^ using a ‘means: Wilcoxon‐Mann‐Whitney test (two groups)' test, with a desired power of 90% and a significance level of 5%. The effect size varied between groups according to the preliminary data acquired during the study. Statistical analysis was performed using OriginPro 9.1 (OriginLab, Northampton, MA).

## Results

### BIOCHEMISTRY

Plasma biochemistry and ammonia concentrations were assessed in all groups of animals (Supporting Table S1). Compared with sham surgery, BDL resulted in a significant increase in plasma alanine aminotransferase and bilirubin (*P* < 0.001), indicating impaired liver function, whereas albumin and total protein concentrations were significantly decreased (*P* < 0.001). Treatment of BDL rats with OP had no effect on these parameters. Rats fed an HA diet had plasma biochemistry similar to that of control rats.

Plasma ammonia concentrations were significantly higher in BDL and HA rats when compared with sham‐operated rats (*P* < 0.001). Treatment of BDL rats with OP lowered plasma ammonia concentration, which was similar to that measured in sham‐operated rats (*P* = 0.3) (Supporting Table S1).

### RELEASE OF LACTATE IN THE CEREBRAL CORTEX IN ANIMAL MODELS OF HE

In cortical slices of sham‐operated rats, enzymatic amperometric biosensors detected tonic lactate efflux of 335 ± 10 μM (n = 18). Recordings from cortical slices of BDL and HA rats showed lower tonic release of lactate of 203 ± 6 μM (*P* = 0.03, n = 18) and 178 ± 8 μM (*P* = 0.005, n = 16), respectively (Fig. 2A). Increasing the permeability of connexin hemichannels by lowering [Ca^2+^]_e_ triggered similar increases in the release of lactate in sham‐operated rats (by 43 ± 3 μM, n = 15), BDL rats (by 38 ± 4 μM; *P* = 0.07, n = 17), and HA rats (by 54 ± 4 μM; *P* = 0.5, n = 16) (Fig. 3A). Hypoxia facilitated the release of lactate in cortical slices of sham‐operated rats (43 ± 3 μM, n = 18) but had no effect on lactate release in slices of BDL rats (1 ± 0.8 μM; *P* < 0.001, n = 18) or HA rats (5 ± 2 μM; *P* < 0.001, n = 16) (Fig. 4A). These results demonstrated impaired tonic and hypoxia‐induced release of lactate in both animal models of HE.

### AMMONIA LOWERING TREATMENT RESTORES CORTICAL LACTATE RELEASE

OP treatment had been shown to decrease systemic and brain ammonia concentrations in BDL rats.^20^ We next found that in our experiments, OP treatment of BDL rats restored tonic (374 ± 11 μM; *P* = 0.4, n = 14) and hypoxia‐induced (32 ± 3 μM; *P* = 0.6, n = 14) lactate release similar to that recorded in cortical slices of sham‐operated rats (Figs. 2A, 3A, and 4A). Direct application of OP on cortical slices of sham‐operated and BDL rats had no effect on lactate release (Supporting Fig. S1D). These results suggest that high ammonia levels are responsible for the reduction in lactate release in the cerebral cortex of BDL rats.

### IMPAIRED HEMICHANNEL FUNCTION UNDERLIES REDUCED CORTICAL LACTATE RELEASE IN ANIMAL MODELS OF HE

In cortical slices of sham‐operated rats, application of hemichannel blockers CBX (n = 9) or NPPB (n = 9) resulted in a significant reduction in lactate release (Fig. 2B,C). Hemichannel blockade had no effect on lactate tone in cortical slices of BDL rats (Fig. 2B,C). However, hemichannel blockade had an effect on lactate release recorded in cortical slices of HA rats (CBX: n = 10; NPPB: n = 8) (Fig. 2B,C). CBX and NPPB reduced lactate tone in cortical slices of BDL rats treated with OP (CBX, n = 10; NPPB, n = 10) (Fig. 2B,C).

In cortical slices of sham‐operated (CBX, n = 8; NPPB, n = 6) and HA rats (CBX, n = 10; NPPB, n = 8), hemichannel blockade using CBX or NPPB abolished or significantly reduced the amount of lactate release facilitated in 0 Ca^2+^ conditions (Fig. 3B,C). A smaller effect of hemichannel blockade on 0 Ca^2+^‐induced release of lactate was observed in cortical slices of BDL rats (CBX, n = 11; NPPB, n = 9) (Fig. 3B,C). In conditions of OP treatment, when tonic lactate release was restored in cortical slices of BDL rats, CBX (n = 7) and NPPB (n = 7) abolished the release of lactate facilitated by 0 Ca^2+^, an effect similar to that observed after application of hemichannel blockers in cortical slices of sham‐operated rats (Fig. 3B,C).

Hypoxia‐induced release of lactate recorded in cortical slices of sham‐operated rats was abolished or dramatically reduced by connexins blockade (CBX, n = 9; NPPB, n = 7) (Fig. 4B,C). CBX or NPPB had no significant effect on the release of lactate induced by hypoxia in cortical slices of BDL (CBX, n = 13; NPPB, n = 9) or HA rats (CBX, n = 10; NPPB, n = 12) (Fig. 4B,C). In cortical slices of OP‐treated BDL rats, the effects of CBX and NPPB were restored. CBX (n = 8) and NPPB (n = 7) effectively abolished hypoxia‐induced lactate release in cortical slices of BDL rats treated with OP (Fig. 4B,C).

Application of the monocarboxylate transporter (MCT) blocker α‐cyano‐4‐hydroxycinnamic acid (4‐CIN) had no significant effect on tonic release of lactate in cortical slices of sham‐operated (n = 5) and BDL rats (n = 5) (Supporting Fig. S2A,B). Hypoxia‐induced lactate release recorded in sham‐operated rats (n = 5) was significantly reduced by 4‐CIN, as reported previously.^13^ In cortical slices of BDL rats (n = 5) the effect of 4‐CIN on hypoxia‐induced release of lactate was found to be smaller (Supporting Fig. S2A,B).

These results demonstrate that connexin hemichannel blockade has no effect on the release of lactate in the cerebral cortex of BDL rats. This implies that the function of hemichannels, which may act as conduits of lactate release,^13^ is already compromised in the brains of these rats. These data also suggest that cortical hemichannel dysfunction in the BDL rats is likely to be due to the actions of ammonia.

### CORTICAL HEMICHANNEL‐MEDIATED DYE LOADING IN ANIMAL MODELS OF HE

Next, membrane channel‐mediated dye loading experiments were performed in cortical slices to confirm hemichannel dysfunction in HE. In cortical slices of sham‐operated rats (n = 10), significant background loading (14.3 ± 0.4 AU) was observed in control conditions when slices were perfused with aCSF containing CBF (Fig. 5A,B). Increasing the permeability of hemichannels by lowering [Ca^2+^]_e_ in the absence of CBF reduced slice fluorescence by 6.4 ± 0.2 AU. The same stimulus applied in the presence of CBF significantly increased fluorescence by 16.9 ± 0.6 AU. Hypoxia‐induced opening of hemichannels in the absence of CBF resulted in dye unloading with fluorescence decreasing by 12.7 ± 0.4 AU. The addition of CBF in conditions of hypoxia increased slice fluorescence by 13.9 ± 0.4 AU (Fig. 5B).

Cortical slices from HA rats (n = 10) displayed background loading (Fig. 5A,B) and 0 Ca^2+^‐induced unloading of 11.2 ± 0.2 AU (*P* = 0.04) and 4.7 ± 0.08 AU (*P* = 0.4), respectively (Fig. 5B). The addition of CBF in 0 Ca^2+^ conditions increased fluorescence by 11.6 ± 0.2 AU (*P* = 0.004), similar to that observed in slices of sham‐operated rats. However, the effect of hypoxia was significantly reduced in slices of HA rats (unloading by 7.6 ± 0.1 AU, *P* < 0.001; loading by 7.8 ± 0.2 AU, *P* < 0.001) (Fig. 5B).

In cortical slices of BDL rats (n = 10), the efficacy of CBF dye loading and unloading was markedly reduced under all conditions (Fig. 5A,B). In cortical slices of BDL rats treated with OP, hemichannel‐mediated CBF dye loading and unloading were similar to that observed in slices of sham‐operated rats (Fig. 5A,B).

Hemichannel blockade with CBX or NPPB markedly reduced CBF dye loading and unloading in cortical slices of sham‐operated rats (Fig. 5C‐D). In cortical slices of BDL and HA rats, CBX and NPPB had no significant effect on dye loading and unloading (Fig. 5C,D). In cortical slices of OP‐treated BDL rats, the effects of CBX and NPPB on CBF dye loading and unloading were similar to that observed in sham‐operated rats. (Fig. 5C,D). Figure 5C,D illustrates changes in fluorescence (ΔFluorescence) following application of the hemichannel blockers, compared with the respective changes in fluorescence recorded in the absence of blockers in slices of the same animal. Negative values show decreases in fluorescence, and positive values illustrate higher fluorescence levels compared with controls.

These data show that the background activity and stimuli‐evoked opening and closure of connexin hemichannels is impaired in BDL rats. The efficacy of CBF dye loading was restored by OP treatment, suggesting that the actions of ammonia are responsible for cortical hemichannel dysfunction in HE.

### CORTICAL CONNEXIN EXPRESSION IN ANIMAL MODELS OF HE

**Figure 6:**
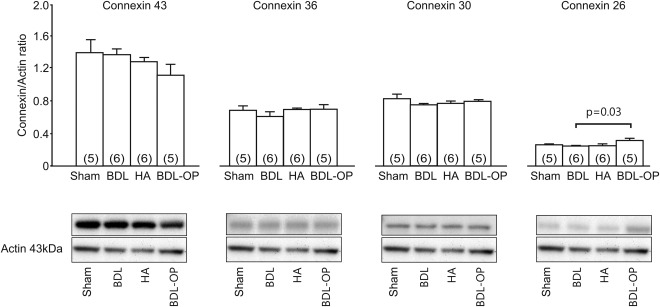
Connexin expression in the cortex is not affected in HE. Top: Summary data illustrating means ± standard error of the densitometry of connexin‐43, connexin‐36, connexin‐30, and connexin‐26 protein expression normalized to the expression of actin in cell lysates of the cerebral cortices of sham‐operated, BDL, HA, and BDL‐OP treated rats. Bottom: Representative immunoblots showing connexin‐43, connexin‐36, connexin‐30, and connexin‐26 protein expression in cerebral cortices of sham‐operated, BDL, HA, and BDL‐OP treated rats. The *P* value indicates the difference in expression level between the BDL and BDL‐OP treated groups.

We next evaluated cortical expression of main astrocytic and neuronal connexins in animal models of HE used in this study. Western blotting was performed on proteins extracted from the cerebral cortices of sham‐operated, BDL, HA, and BDL‐OP treated rats (Fig. 6). No differences in cortical connexin‐43, connexin‐36, or connexin‐30 expression was observed between sham‐operated, BDL, HA, and OP‐treated BDL rats. A small increase in connexin‐26 expression (*P* = 0.03) was observed in BDL‐OP treated rats compared with BDL rats (Fig. 6). Expression of MCT‐1 was similar in all experimental groups (Supporting Fig. S2C).

## Discussion

Brain information processing requires a constant and sufficient supply of oxygen and metabolic substrates. Astrocytes represent an important source of lactate, which contributes to the extracellular pool of readily available metabolic substrates taken up by neurons to fuel their activity.^31^ Although it was thought previously that lactate transport across the cell membranes was achieved solely through the operation of MCTs, a recent study^13^ demonstrated that connexin hemichannels are equally important conduits of lactate release.

In animal models of and patients with ALF, an increase in brain lactate concentration has been reported.^32^ Concentrations of lactate in the cerebrospinal fluid were also found to be elevated in patients with cirrhosis, but only in severe cases of HE.^33^ The BDL and HA rats used in our experiments are models of minimal HE.^34^ In contrast to the existing evidence suggesting that brain lactate concentrations are increased in ALF,^35^ our experiments demonstrated that the development of HE in rats is associated with a significant reduction in tonic and stimulated release of lactate in the cerebral cortex. Blockade of connexin hemichannels was found to be effective in reducing lactate release in sham‐operated and HA rats but was ineffective in BDL rats, suggesting that the reduction in hemichannel‐mediated lactate release in BDL rats is due to a combination of pathological factors (e.g., inflammation, oxidative stress).

Increased lactate production by astrocytes appears to be essential for the recovery of synaptic function during reoxygenation after hypoxia.^36^ We found that hypoxia‐induced lactate release was significantly reduced in the cerebral cortex in BDL and HA rats compared with control rats, and was unaffected by the connexin blockers, indicating hemichannel dysfunction. The observed decrease in extracellular lactate is likely due to impaired release from astrocytes, although increased neuronal activity and therefore lactate consumption cannot be excluded. The results reported by Bosoi et al.^37^ showing higher total brain lactate in BDL rats using NMR spectroscopy seem to contradict our data. Bosoi et al. suggested that increased lactate contributes to the pathogenesis of brain edema (cytotoxic) and may imply that the observed increase in total brain lactate is due to its intracellular accumulation. If the rate of lactate production and glymphatic clearance^38^ are not affected, intracellular retention of lactate would explain higher concentration of this metabolite as measured by NMR spectroscopy^39^ and would be in full agreement with our data showing impairment of hemichannel‐mediated release in HE.

High concentration of ammonia can potentially generate significant pH changes, which can have various effects on many pH sensitive membrane channels, including hemichannels. The pH sensitivity of hemichannels is known as the chemical gate, which is the phenomenon of hemichannel blockade when intracellular pH (pH_i_) decreases.^39^ This provides one potential mechanism that may be responsible for impaired hemichannel‐mediated lactate release in HE.

We also examined hemichannel functionality using the dye‐loading method. Some differences between the data obtained using biosensor recordings and this technique could be due to the fact that CBF is not identical to the molecular structure and size of lactate. Additionally, lactate could be released through specific connexin hemichannels, whereas CBF is small enough to pass through the majority of hemichannels expressed by both astrocytes and neurons. Whereas hypoxia may predominantly affect astroglial hemichannels, the rest of the experimental conditions are not “cell specific” and conclusions regarding specific cell types involved cannot be drawn from the results obtained in dye‐loading experiments.

The experimental stimuli used (0 Ca^2+^ and hypoxia) are known to increase the permeability of hemichannels, possibly by affecting various protein bonds resulting in conformational changes.^28^ Dye‐loading experiments clearly demonstrated significant reduction in fluorescent dye uptake and release in cortical slices of BDL and HA rats compared with sham‐operated rats (the differences were more profound when hypoxia was used as a stimulus), suggesting reduced bidirectional permeability of hemichannels in these animal models of HE. Hemichannel blockade had little effect on fluorescent dye uptake in BDL and HA rats, providing additional evidence that the function of these channels is already compromised. Ammonium ions may cause structural alterations to the connexin proteins by interacting with various amino acid side chains, which could affect gating of the channel. However, because hemichannels have a relatively short life cycle and are recycled frequently, the observed changes in hemichannel functionality appear to be reversible with OP treatment.

In chronic liver disease, hyperammonemia is believed to impair mitochondrial function and induce astroglial dysfunction, which is associated with altered neurotransmitter recycling leading to neuronal damage.^40^ Ammonia may also interfere with cell energy metabolism in several ways. There is recent evidence that in astrocytes, ammonia may acutely divert the flux of pyruvate to lactate production, contributing to the net aerobic lactate production.^7^ However, the effects of chronic ammonia exposure on astrocytes are unknown. We investigated the role of ammonia by treating BDL rats with OP, a drug known to lower systemic and brain ammonia.^20^ OP treatment improved the neurochemical phenotype of BDL rats by restoring the tonic and stimulated hemichannel‐mediated lactate release. Furthermore, hemichannel blockade became effective after OP treatment, suggesting that ammonia is indeed responsible for hemichannel dysfunction observed in this model.

Cytotoxic brain edema observed in BDL rats is attenuated by ammonia‐lowering treatments such as the one used in the present study.^20^, ^41^ The effect of cell swelling on hemichannel function is poorly understood. Ye et al.^42^ showed that astrocytes obtained from connexin‐43 knockout animals developed cell swelling as efficiently as wild‐type animals when exposed to a hypotonic solution, suggesting that hemichannels do not play a significant role in this process despite evidence to the contrary.^43^

We also examined the expression profile of key astroglial and neuronal connexins in the animal models of HE used in this study. No significant differences in connexin hemichannel protein expression profile were observed, suggesting that HE is associated with altered hemichannel function but not with changes in connexin expression. The up‐regulated expression of connexin‐26 observed in BDL‐OP treated rats is not prominent enough to explain the marked improvement observed in the lactate measurements and dye‐loading experiments. Additionally, we did not observe any changes in the expression of the main astroglial lactate transporter MCT‐1 in the models of HE used in this study. No effect of the MCT blocker 4‐CIN on lactate release was observed in cortical slices of sham‐operated or BDL rats. Hypoxia‐induced lactate release in sham‐operated rats was found to be significantly reduced by the application of 4‐CIN, as reported previously.^13^

Depletion of lactate as one of the key readily available metabolic substrates may have important neurological consequences, particularly in patients with advanced cirrhosis given the fact that these patients display evidence of cerebral vasoconstriction,^44^ which is associated with impaired cerebral autoregulation, an important mechanism that ensures constant cerebral blood flow.^45^ The clinical consequences of this may be relevant during liver transplantation, where further reductions in cerebral blood flow have been observed during the anhepatic phase of transplantation and may contribute to posttransplantation neurologic dysfunction.^46^ Evidence for critical reduction in cerebral oxygenation was obtained in the majority of patients with acute‐on‐chronic liver failure who had poor neurologic outcome, supporting the hypothesis that the brain energy metabolism is critically compromised in cirrhosis and that further perturbations as demonstrated in the present study may be clinically deleterious.^47^ Our data indicating an impaired hemichannel‐mediated lactate release during tissue hypoxia, in combination with these observations, could help explain the severe neurological manifestations in patients with HE. Because ammonia is central in causing this dysfunction, the potential clinical implications involve the use of ammonia‐lowering treatments as a main therapeutic strategy, as well as attempts to increase cerebral oxygenation to preserve neuronal function.

In conclusion, the results of the present study suggest that HE is associated with central nervous system hemichannel dysfunction, with ammonia playing a key role. The data provide evidence of a potential neuronal energy deficit due to impaired hemichannel‐mediated lactate transport between astrocytes and neurons as a possible mechanism underlying the pathogenesis of HE.

Author names in bold designate shared co‐first authorship.

## Supporting information

Additional Supporting Information may be found at onlinelibrary.wiley.com/doi/10.1002/hep.29031/suppinfo

Supporting Information
