# Supplementary material for: Ammonia mediates cortical hemichannel dysfunction in rodent models of chronic liver disease
Source: Hepatology. 2017 Mar 7;65(4):1306–18. doi: 10.1002/hep.29031 (PMC5396295; doi:10.1002/hep.29031)
Supplement: Supplementary file 1 — Supporting Information [file HEP-65-1306-s001.pdf]

SUPPLEMENTARY MATERIAL

Supplementary Figure 1: Response time, principles of lactate biosensor operation and the effect of OP on lactate detection system. A) A schematic drawing of the lactate biosensor assembly showing the enzymatic biolayer, which surrounds the tip of the platinum (Pt) wire. **B)** Enzymatic reaction taking place in the biolayer of the sensor. **C)** Expanded portion of the lactate calibration trace illustrating the response characteristics of the lactate biosensor. Note that the sensor responds immediately when lactate solution is starting to enter the calibration chamber. **D)** Raw traces illustrating lactate biosensor current responses to lactate (100 μM) when calibrated in control artificial cerebrospinal fluid (aCSF), and in the presence of ornithine phenylacetate (OP, 1:50 dilution of the stock solution of 0.1g/ml; concentration applied is similar to the concentration estimated to be reached in a 300 g rat following IP injections). *Bottom*: Representative trace from the recordings obtained in the cerebral cortex of a BDL rat showing the effect of OP on lactate tone. Grey shading indicates period of drug application.

Supplementary Figure 2: Monocarboxylate transporter (MCT) protein expression and functionality in cortical slices of sham-operated and BDL rats. A) Summary data illustrating the effect of MCT blocker, 4-CIN (250 Mm, Sigma) on tonic release of lactate (expressed as % change from the baseline) and release of lactate facilitated in response to tissue hypoxia and 0 $[Ca^{2+}]_e$ (expressed as the % of the amount of lactate released in response to hypoxia/0 $[Ca^{2+}]_e$ in the absence of 4-CIN) in cortical slices of sham-operated,

BDL, HA and BDL-OP treated rats. *p* values indicate differences between responses recorded in the absence and presence of 4-CIN. **B)** Representative recordings of lactate biosensor current showing the effect of 4-CIN on tonic as well as hypoxia and 0 $[Ca^{2+}]_e$ -induced release of lactate. **C)** Summary data illustrating means ± SE of the densitometry of MCT-1 protein levels, mainly expressed in astrocytes (Chemicon International, 0.5 µg/mL) normalized to the expression of Actin, in cell lysates of the cerebral cortices of sham-operated, BDL, HA and BDL-OP treated rats. *Bottom*: Representative immunoblots showing MCT-1 protein expression in cerebral cortices of sham-operated, BDL, HA and BDL-OP treated rats.

Supplementary Table 1: Plasma biochemistry.

Bile duct ligated (BDL), hyperammonemic (HA), ornithine phenylacetate treated BDL rats (BDL-OP)*; ALT*, alanine aminotransferase.

Data expressed as means±SEM, ** $p<0.001$ compared to sham group using one-way ANOVA.

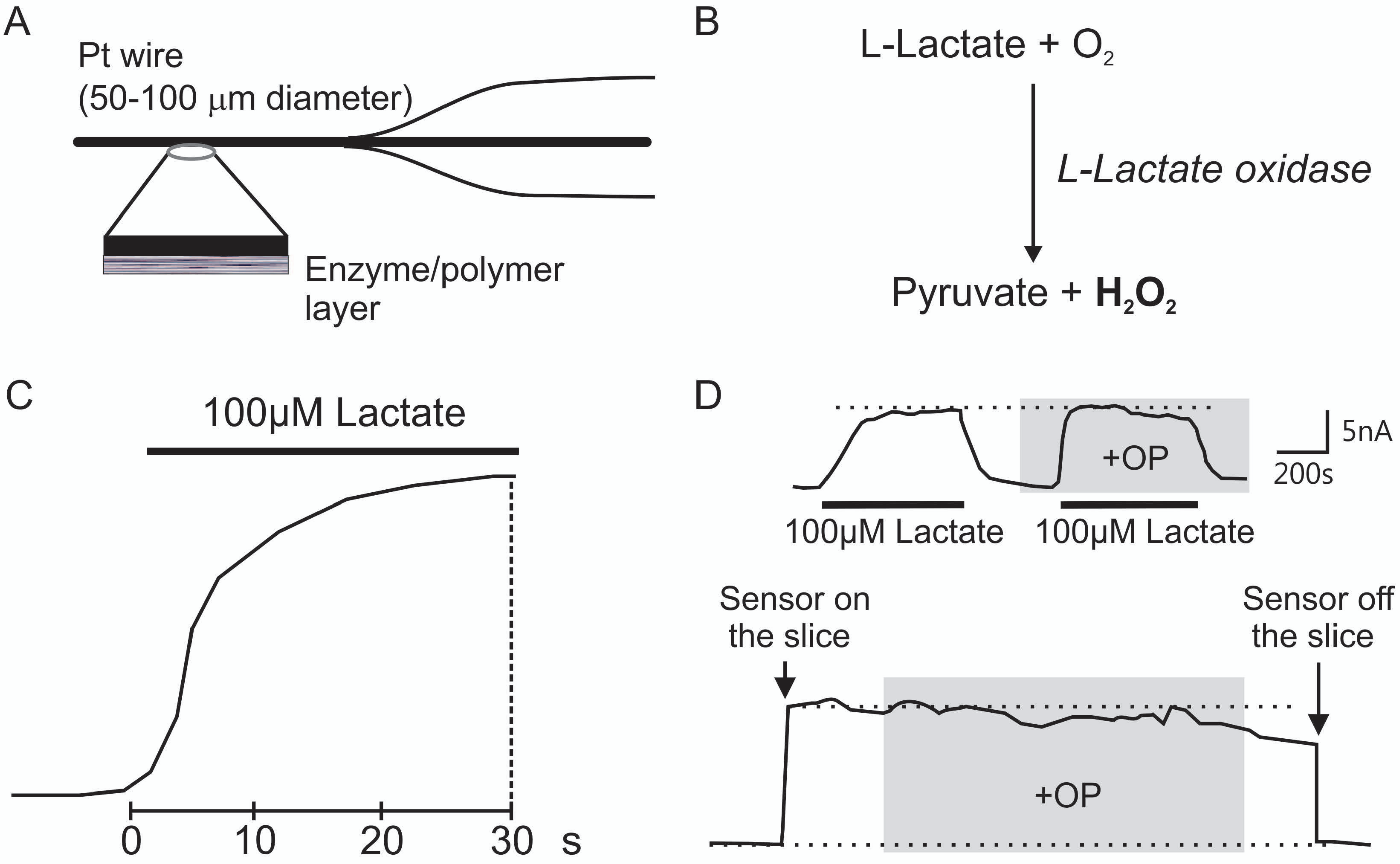
A
Pt wire
(50-100 μm diameter)
Enzyme/polymer
layer
B
L-Lactate + O2
L-Lactate oxidase
Pyruvate + H2O2
C
100μM Lactate
0
10
20
30
s
D
5nA
200s
+OP
100μM Lactate
100μM Lactate
Sensor on
the slice
Sensor off
the slice
+OP

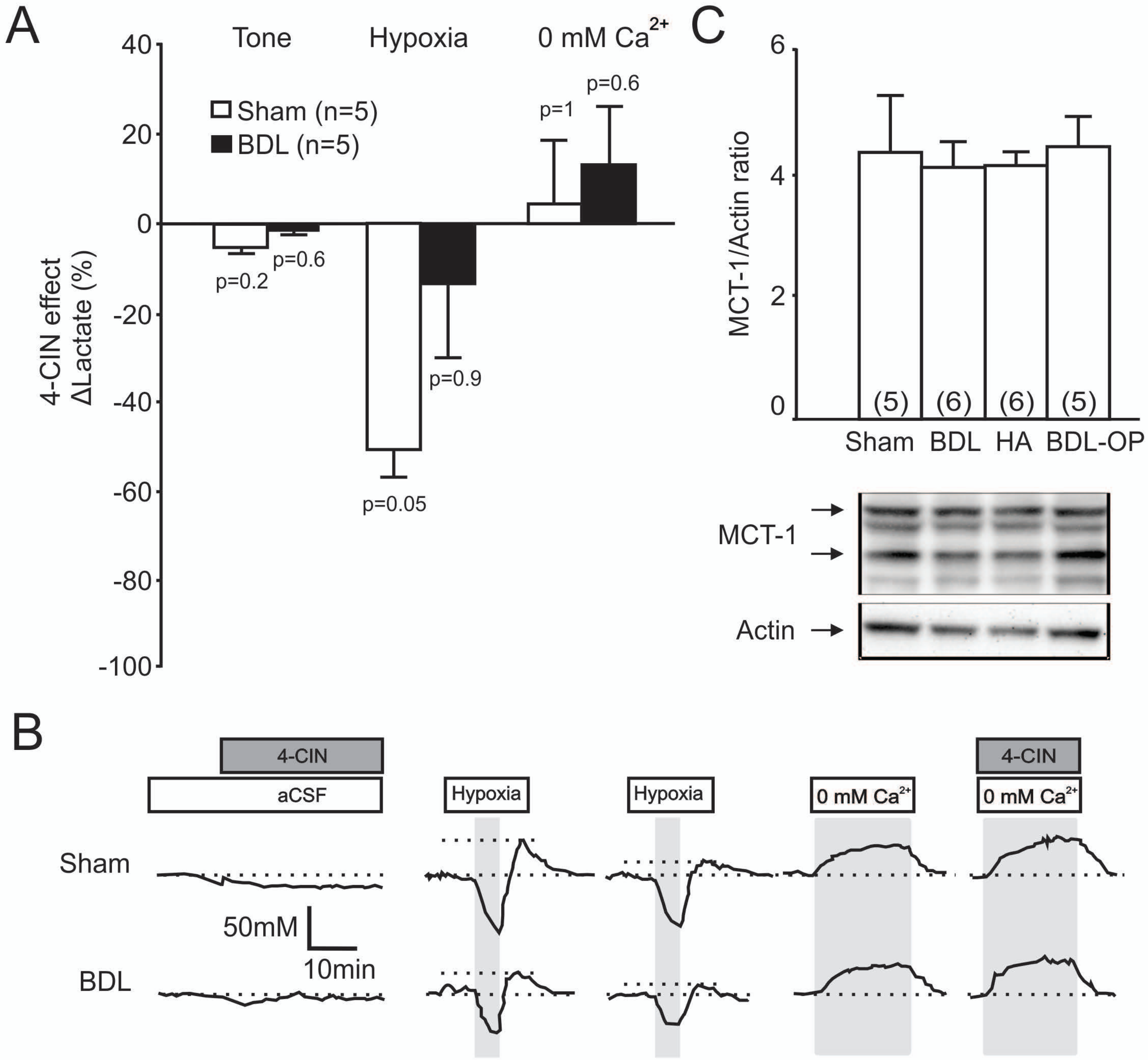
A
Tone
Hypoxia
0 mM Ca2+
Sham (n=5)
BDL (n=5)
4-CIN effect
ΔLactate (%)
40
20
0
-20
-40
-60
-80
-100
p=0.2
p=0.6
p=0.05
p=0.9
p=1
p=0.6
C
MCT-1/Actin ratio
6
4
2
0
(5)
(6)
(6)
(5)
Sham
BDL
HA
BDL-OP
MCT-1
Actin
B
4-CIN
aCSF
Hypoxia
Hypoxia
0 mM Ca2+
4-CIN
0 mM Ca2+
Sham
50mM
10min
BDL

Parameters	Sham	BDL	HA	BDL+OP
**N numbers**	**22**	**26**	**22**	**14**
**Ammonia, µmol/L**	56±3	141±4**	121±9**	60±2
**Albumin, g/L**	35±4	23±0.4**	30±0.8	23±1
**Total protein, g/L**	50±0.3	38±0.5**	51±3	40±1
**Bilirubin, µmol/L**	5±0.5	205±3**	4±0.6	161±6
**ALT, U/L**	11±0.4	130±2**	17±4	87±4
